# Incidence and Risk Factors of Elder Mistreatment in the Community: A Longitudinal Population-Based Study

**DOI:** 10.1093/geroni/igab046.335

**Published:** 2021-12-17

**Authors:** David Burnes, David Hancock, John Eckenrode, Mark Lachs, Karl Pillemer

**Affiliations:** 1 University of Toronto, Toronto, Ontario, Canada; 2 Weill Cornell Medicine, New York City, New York, United States; 3 Cornell University, Ithaca, New York, United States

## Abstract

Prior population-based elder mistreatment (EM) risk factor research has focused on problem prevalence using cross-sectional designs, which cannot make causal inferences between proposed risk factors and EM or discern existing cases from new cases entering the population. This study sought to estimate the incidence of EM and identify risk factors for new cases. It is a ten-year prospective, population-based cohort study with data collected between 2009 (Wave 1) and 2019 (Wave 2). Based on Wave 1 random, stratified sampling to recruit English/Spanish-speaking, cognitively intact, community-dwelling older adults (age ≥ 60) across New York State, this study conducted computer assisted telephone interviews (CATI) with 628 respondents participating in both Wave 1 and Wave 2 interviews (response rate=60.7%). Ten-year EM incidence was regressed on factors related to physical vulnerability, living arrangement, and socio-cultural characteristics using logistic regression. Ten-year incidence rates included overall EM (11.4%), financial abuse (8.5%), emotional abuse (4.1%), physical abuse (2.3%), and neglect (1.0%). Poor self-rated health at Wave 1 significantly predicted increased risk of new Wave 2 overall EM (odds ratio [OR]=2.8), emotional abuse (OR=3.67), physical abuse (OR=4.21), and financial abuse (OR=2.8). Black older adults were at significantly heightened risk of overall EM (OR=2.61), specifically financial abuse (OR=2.8). Change from co-residence (Wave 1) toward living alone (Wave 2) significantly predicted financial abuse (OR=2.74). Healthcare visits represent important opportunities to detect at-risk older adults. Race is highlighted as an important social determinant for EM requiring urgent attention. This study represents the first longitudinal, population-based EM incidence study.

